# Handheld robotic device for endoscopic neurosurgery: system integration and pre-clinical evaluation

**DOI:** 10.3389/frobt.2024.1400017

**Published:** 2024-06-05

**Authors:** Emmanouil Dimitrakakis, George Dwyer, Nicola Newall, Danyal Z. Khan, Hani J. Marcus, Danail Stoyanov

**Affiliations:** ^1^ Wellcome/EPSRC Centre for Interventional and Surgical Sciences (WEISS), University College London, London, United Kingdom; ^2^ Panda Surgical Limited, London, United Kingdom; ^3^ National Hospital for Neurology and Neurosurgery, London, United Kingdom

**Keywords:** medical robotics, handheld robotics, robotic neurosurgery, endoscopic neurosurgery, endonasal approach

## Abstract

The Expanded Endoscopic Endonasal Approach, one of the best examples of endoscopic neurosurgery, allows access to the skull base through the natural orifice of the nostril. Current standard instruments lack articulation limiting operative access and surgeon dexterity, and thus, could benefit from robotic articulation. In this study, a handheld robotic system with a series of detachable end-effectors for this approach is presented. This system is comprised of interchangeable articulated 2/3 degrees-of-freedom 3 *mm* instruments that expand the operative workspace and enhance the surgeon’s dexterity, an ergonomically designed handheld controller with a rotating joystick-body that can be placed at the position most comfortable for the user, and the accompanying control box. The robotic instruments were experimentally evaluated for their workspace, structural integrity, and force-delivery capabilities. The entire system was then tested in a pre-clinical context during a phantom feasibility test, followed up by a cadaveric pilot study by a cohort of surgeons of varied clinical experience. Results from this series of experiments suggested enhanced dexterity and adequate robustness that could be associated with feasibility in a clinical context, as well as improvement over current neurosurgical instruments.

## 1 Introduction

Due to its delicate subject matter and challenging operations, neurosurgery has always been in need of adapting new techniques and technologies Marcus et al. (2015). A neurosurgical procedure that could especially benefit from the use of such technologies is Endoscopic Endonasal Transsphenoidal Surgery (EETS), a minimally invasive neurosurgical technique that is performed via an anterior sphenoidotomy and aims to remove sellar and parasellar lesions with the use of an endoscope and rigid instruments Cappabianca et al. (2004). In recent years, there has been an increased interest in the Expanded Endoscopic Endonasal Approach (EEEA) that expands the EETS areas of interest to include the regions from the cribriform plate of the anterior cranial fossa to the foramen magnum in the anteroposterior plane Dehdashti et al. (2009). Although a promising alternative to transcranial approaches, that require craniotomies and brain retraction, the EEEA comes with its limitations. The lack of instrument articulation, combined with the constrained operative space of the nasal channel, are some of the biggest operative challenges Marcus et al. (2014).

Robotic-assisted minimally invasive surgery (RAMIS) allows for increased instrument articulation, and thus surgeon dexterity, even in operative workspaces with restricted access Peters et al. (2018). Thus, it is deemed as a suitable potential solution for the EEEA. In recent years, concentric tube robots (CTR) have been developed for this operation since their miniature structure allows for increased dexterity and reachability in constrained spaces. One such example is the robot developed in Burgner et al. (2011), where a tele-operated robotic system with miniature, tentacle-like tool shafts for bimanual tele-operated endonasal skull base surgery is developed and evaluated for its feasibility in a pilot cadaver study. A demonstration that concentric tube robots can provide many of the benefits of robotic technology to skull-base surgery was conducted in Swaney et al. (2015), where a CTR system was used to remove simulated pituitary tumors from a skull phantom. This sentiment is expanded in Wang et al. (2020), with the design, analysis, and experimental evaluation of a three-arm CTR system suggesting clinical feasibility and capability.

Despite some of the possible advantages of concentric tube robots, there are still concerns about the distal-end dexterity of these manipulators and their force-delivery capabilities Li et al. (2017). Thus, they are not the only robotic systems developed for the endonasal approach. In Wei et al. (2021), a novel continuum design is fabricated with laser cutting and by deploying a superposition methodology for bending parameters and thus joint configuration determination. Finally, a miniaturized articulated robotic forceps with four Degrees-of-Freedom (DoF) made of a compliant structure is presented in Bandara et al. (2022), and evaluated for its output and grasping force, repeatability, and robustness within the context of endonasal surgery.

Articulation is often used as a means to improve tissue manipulation tasks, such as tumour removal, but it can also be useful for visualization of the constrained anatomies in minimally invasive neurosurgical procedures. This is why in Eastwood et al. (2020), a steerable instrument for neuroendoscopy is presented, with a wrist being incorporated into a standard neuroendoscopic instrument via use of a notch-tube joint design. Finally, in Phelan III et al. (2022), the design and development of an MRI-driven endoscope leveraging the high external magnetic field of an MR scanner for heat-mitigated steering within the ventricular system of the brain is proposed.

What these systems have in common, is their actuation and control means. They deploy a tele-operated control methodology where the surgeon is manipulating the robotic device from a master-console. While tele-operation has traditionally been the most common manipulation means for robotic systems aimed to enhance surgery, including the EETS and the EEEA, recently, handheld robotic devices have started gaining popularity due to offering some advantages over these bulkier and more complex remotely controlled systems Marcus et al. (2013). Handheld robotic instruments have a smaller footprint and can be associated with smaller purchasing and maintenance costs. Additionally, they can be easily integrated into the surgical work flow due to their ability of quick instrument change and due to their resemblance to standard equipment, they can decrease the surgeon’s learning curve Payne and Yang (2014). Especially during the EEEA, a tele-operated system would significantly increase the operating complexity, due to the need for frequent tool changes.

The field of handheld robotic surgical instruments is densely populated with devices in various developmental stages. In Leite et al. (2016), a new hand-held robotic device for laparoscopy is developed to overcome common laparoscopic surgery problems such as the restricted mobility inside the human body. The aim of this study was to ascertain the influence of the handheld robotic instrument on laparoscopic skills performance of 2 different groups, naive and expert. A similar design is implemented in Feng et al. (2020), where a handheld robotized needle holder was compared with a conventional tool with the aim of clearly demonstrating the learning curve of the robotic instrument.

As with tele-operated systems, CTRs make their appearance in more compact form-factors too. The first fully handheld CTR capable of 6-DoF motion was introduced in Girerd and Morimoto (2020). This lightweight device is controlled through an interface that decouples the displacements of the tip to in-plane motions. While a potential improvement over standard instruments, controlling concentric tubes with such a contained form factor make the design and implementation of handheld CTRs especially difficult. This is why a different design philosophy is followed in Hernández-Valderrama et al. (2022), where a steerable robotic instrument with a curved sliding-joints design that articulates the distal tip in two additional DoF relative to the instrument shaft is presented, and in Wang X. et al. (2022), where the authors develop a hybrid-structure for sinus surgery with a novel robotic manipulator and a compact, light hand-held actuation system for flexible endoscopy.

Other than soft-tissue surgery, handheld devices with enhanced articulation have also been introduced in surgeries that aim in treating bony-tissue. In Payne et al. (2015), a robotic device with a flexible manipulator with intelligent trajectory following for athroscopic interventions is developed and evaluated for different bending conditions alongside its overall robustness. A cadaveric study was also performed to demonstrate the potential clinical value of the device. Underlining the need for added articulation when dealing with bony tissue, the authors of Wang Y. et al. (2022) developed a handheld steerable surgical drill for dexterous bone-work in confined spaces. The core component of this drill is a novel miniaturized joint module, composed of a structurally simple tendon-driven geared rolling joint and a double U-joint.

Both the fields of neurosurgical robotics, as well as handheld robotics for various other disciplines, are populated with exciting works. However, there are still some unmet needs in their design and implementation in order to be deployed in EETS and EEEA. Most tele-operated robots aimed for endonasal approaches would potentially increase the operative setup time, and the duration of the operation due to the various instrument-changes required during endoscopic neurosurgery. Similarly, handheld robots aimed for other disciplines are often too big for the confined spaces of the endonasal approach, they deploy control interfaces that are not suitable for the operative workspace, and even when neither situation applies and they are designed for the EEEA, they still do not implement quick instrument change mechanisms.

This work presents a novel handheld robotic instrument for endoscopic neurosurgery. It expands on previous work on miniature end-effectors and handheld controllers, with the aim to develop an advanced functional robotic prototype. The system is comprised of a series of interchangeable articulated spherical-joint tendon-driven 3 *mm* end-effectors that expand the operative workspace and enhance the surgeon’s dexterity, paired with an ergonomically designed handheld controller that is indifferent to hand-size and handedness, and does not require the shaft to be resting against a trocar-port making it suitable for the cylindrical access pathways of endoscopic neurosurgery. This first-of-its-kind robotic instrument for neurosurgery was evaluated during a series of engineering tests, before being put through a round of pre-clinical studies. Results from both testing cycles suggested feasibility within a clinical context, as well as improvement over current neurosurgical instruments.

## 2 Materials and methods

In previous work, a 3.6 *mm* miniature end-effector for endoscopic neurosurgery was developed Dimitrakakis et al. (2020). This manipulator deployed a tendon-driven spherical joint design and was evaluated for its extended workspace and structural integrity. Preliminary work on pairing this end-effector with an appropriate handheld controller included the design and fabrication of two concept handle prototypes that were compared in terms of performance and ergonomics Dimitrakakis et al. (2022).

**FIGURE 1:**
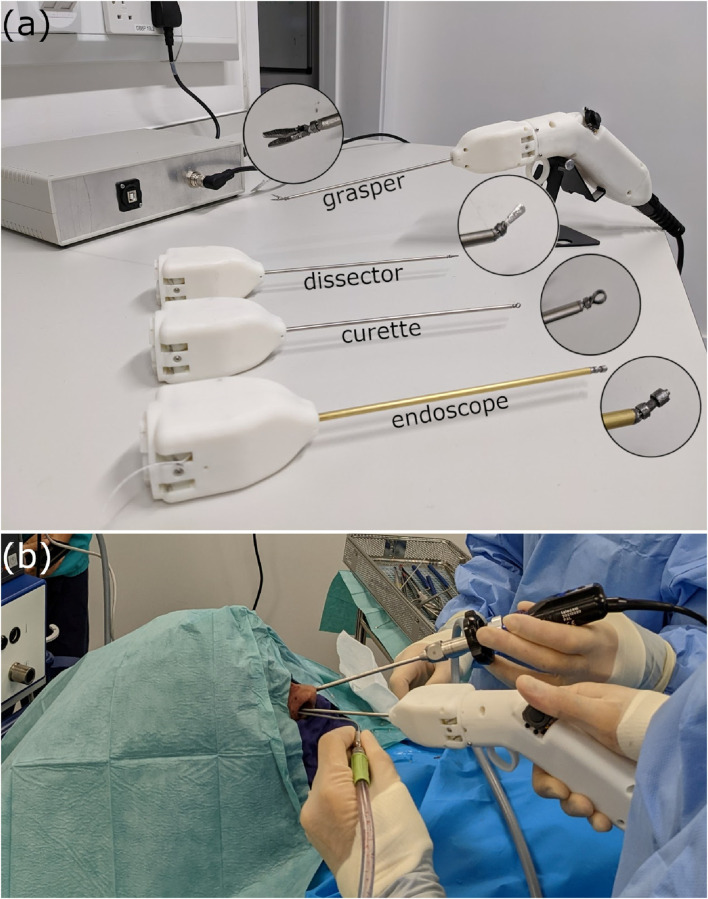
**(A)** The handheld robotic instrument deploying a grasper, alongside three other articulated tools; a flat dissector, a ring-curette, and an endoscope, and **(B)** the handheld device used in a simulated cadaveric operation of the endonasal approach.

That work laid the foundation for the development of this handheld robotic system. The system is comprised of a series of detachable 3 *mm* end-effectors, an ergonomically designed handheld controller with an adjustable joystick-and-trigger interface weighing 247*g*, and lastly the handheld controller is connected to a control box containing the power supply and microcontroller. The full system can be seen in Figure 1.

### 2.1 Interchangeable end-effectors

The robotic end-effector located at the distal end of our novel handheld robotic instrument was initially introduced in previous work Dimitrakakis et al. (2020). While promising results from that preliminary study suggested that introducing a miniature robotic end-effector could enhance surgical capability; it also identified areas which required further development, namely, the workspace of the end-effector, and more importantly its structural integrity.

**FIGURE 2:**
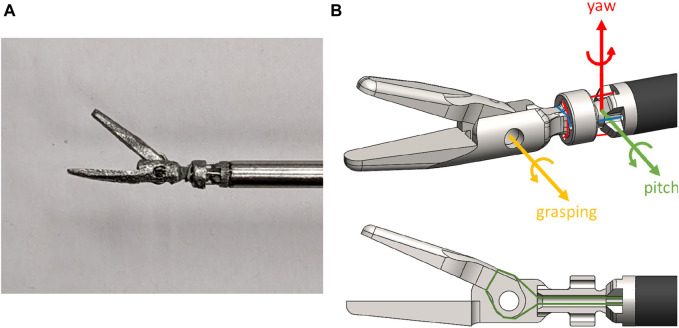
**(A)** The miniature grasper end-effector, and **(B)** the tendon-routing of the 3 DoF, alongside the coordinate system of the joint DoF.

The newly developed end-effector has an overall diameter of 3 *mm* and deploys a 2 DoF tendon-driven spherical joint that allows movement in the pitch and yaw axes around a rolling surface. The roll axis motion around its rolling surface is restricted by the tendons and can be compensated by the surgeon’s hand movement. The third DoF of the end-effector is the opening and closing of the grasper. This articulated grasper was fabricated in stainless steel with additive manufacturing techniques, namely, direct metal laser sintering (3DSystems, USA), and was laser-welded onto a 3 *mm*-diameter stainless steel shaft. The fully-assembled grasper is shown in Figure 2A.

The tendons used to antagonistically control the end-effector were 0.3 *mm*-diameter stainless steel wire rope (ENGELMANN Drahtseilfabrik GmbH, DE). A single tendon was used per DoF, the tendon was looped around the spherical joint-body and then fixed to the body with adhesive to avoid slippage during pre-tensioning. To actuate the grasper DoF, the tendons were passed through a hollow channel in the middle of the spherical joint body and then looped and fixed about the axis of the grasper. Figure 2B details the tendon-routing for all 3 DoF of the articulated grasper.

**FIGURE 3:**
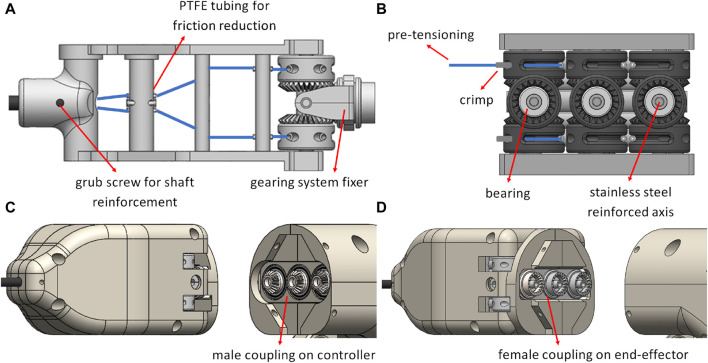
**(A)** The tendon-routing system for a single DoF with the other two DoF tendons following similar routing paths, **(B)** the differential gearing system, highlighted in grey, with tendons for a single DoF, **(C)** the coupling on the handheld controller side, and **(D)** the coupling on the end-effector side.

To route the tendons from the distal end of the tool, where the robotic end-effector is located, to the proximal end of the tool, at the point where it couples with the handheld controller, a routing sub-system was used. The tendons were terminating on the end-effector socket-joint as shown in Figure 2B. They were then routed through the stainless steel shaft of the tool and additionally through a series of 3 contact points as shown in Figure 3A, before passing through geared capstans. The tendons were pre-tensioned at 15*N* and crimped. Finally, the two capstans were rotating in opposite directions with a perpendicular bevel gear. The differential gearing system with the tendon pre-tensioning mechanism is evident in Figure 3B.

**FIGURE 4:**
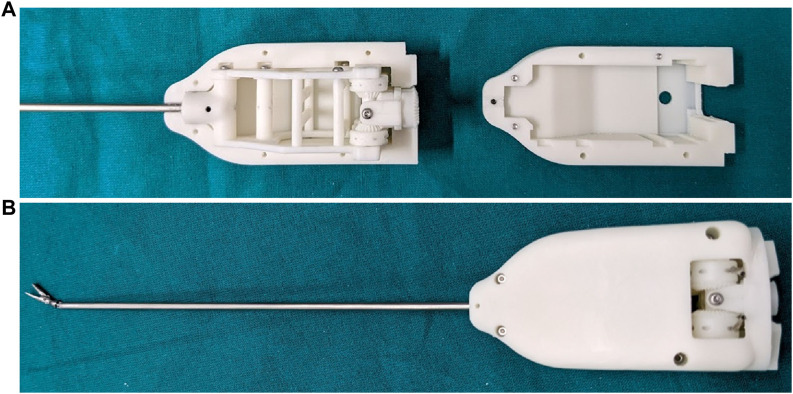
**(A)** The routing system inside an open end-effector casing, and **(B)** the fully assembled grasper end-effector.

To couple the end-effector with the handheld controller, a male-to-female connection is used between the gears that are placed on the motors shafts, and the bevel gears that are part of the differential gearing system. The coupling on both the handheld controller and end-effector sides are depicted in Figures 3C,D. The end-effector and the handheld controller coupling is secured with two alignment pins, one on either side of the instrument. The routing system inside an open casing, as well as a full assembly are shown in Figure 4.

**FIGURE 5:**
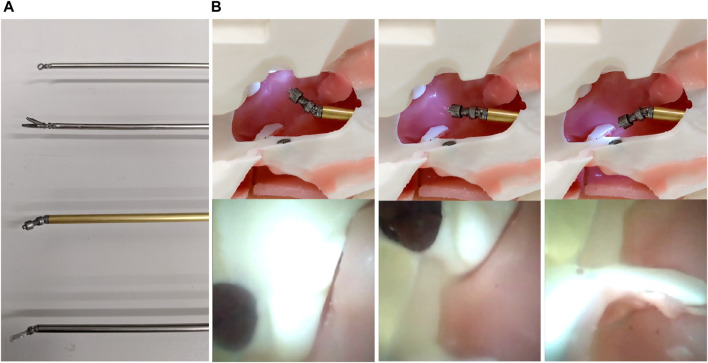
**(A)** (Top to bottom) The articulated end-effectors, namely, the ring-curette, the grasper, the endoscope, and the spatula dissector, and **(B)** Top row—The articulated endoscope inside a pituitary anatomy phantom at three different angles. Bottom row—the accompanying views from the camera. The dark spot seen in the top row of pictures is a magnet used for the phantom assembly, not to be confused with the simulated tumor evident in the bottom row.

To cover a larger set of interchangeable neurosurgical instruments, we amended the grasper end-effector design to enhance other standard instruments with robotic articulation. Namely, these instrument end-effectors were a ring-curette, a spatula dissector, and an endoscope. These instruments do not deploy a third DoF, but rather only need pitch and yaw articulation, with the endoscope specifically also requiring an expanded hollow working channel with a diameter of 1.8 *mm* for the 1.66 *mm*-diameter miniature camera MD-V1000LH-120 UVC (MISUMI Electronic Corporation, TW) to pass through. This increased the overall diameter of the endoscope to 4 *mm*. The alternative end-effectors, alongside the articulated endoscope and its expanded field of view, are found in Figure 5.

### 2.2 Ergonomic handheld controller

**FIGURE 6:**
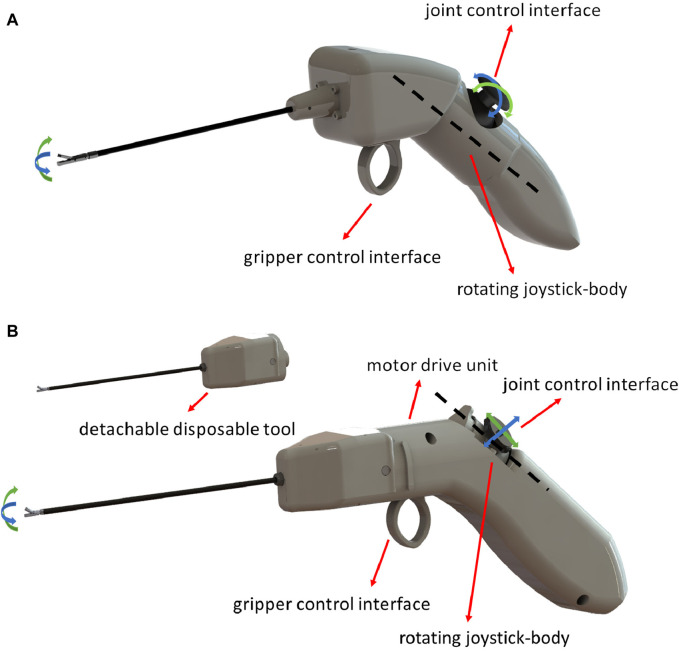
Renderings of **(A)**. The concept handheld design suggested in Dimitrakakis et al. (2022) tested in simulation, and **(B)** The finalised handheld instrument design after electronics, motors, and end-effectors were incorporated to offer functionality.

To control the end-effector, we aimed to build a handheld controller that would be ergonomically designed to not cause the surgeon strain or fatigue, while also being easy to use and associated with small learning curves. In previous work, we have attempted to design two drastically different handheld controller concepts covering a wide array of ergonomic design suggestions found in literature Dimitrakakis et al. (2022), Dimitrakakis et al. (2021). The two design concepts, as well as a standard instrument, were compared for their efficacy and ergonomics during a pre-clinical randomised controlled trial, with the design shown in Figure 6A proving superior.

According to literature suggestions Dimitrakakis et al. (2022), this finger-actuated handheld controller should cater to different hand sizes, and include a large palmar grip surface that would allow a comfortable and robust grip. To achieve the former ergonomic requirement, we designed a rotating joystick-body that could be placed at a position most comfortable for the surgeon, depending on their hand size and handedness.

The handle with the shaft should maintain a 45^*°*^ angle to avoid wrist-strain when maintaining it, and to make the control instinctively easy to adopt, the thumb should control the robot-joints, and the index-finger should operate the robotic grasper. After our concept design was preliminarily validated in previous work, our next developmental step was to incorporate motors and electronics to turn it into a fully-functional handheld robotic controller. Figure 6B showcases a rendering of the finalised instrument design.

**FIGURE 7:**
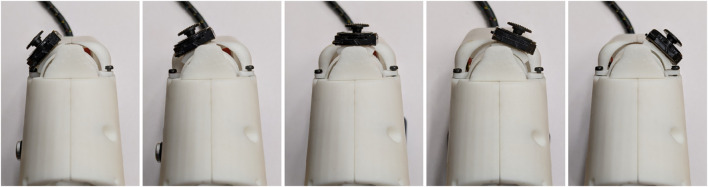
The rotating joystick-body in its 5 discrete positions with a rotating step of 15^*°*^.

The previously suggested rotating joystick-body, a core element of the ergonomics of the device, was incorporated with the use of a translational 2-axis joystick alongside a compact rotating platform accommodating the motor placement inside the device. To fix the rotating platform in the desired position, we introduced a multi-pin that fixes it in place. The rotating joystick-body in its 5 discrete positions is depicted in Figure 7.

The handheld controller is also 3D-printed with resin, similar to the end-effector casings, and the motor drive unit housed inside the handle consists of 3 lightweight and compact motors. These motors are brushed DC-motors with an outer diameter of 8 *mm* and a 2.34 *mm* shaft diameter (FAULHABER, DE). Gears are fixed on the shafts of the motors that are then coupled with the gears located on the proximal end of the end-effector tools as previously described in Section 2.1. That way the motor movement is transmitted over to the robot joints. Finally, other than the motors, the handle houses the 2-axis joystick for joint control and a 10*K*Ω trimmer potentiometer for trigger control.

### 2.3 Control box

**FIGURE 8:**
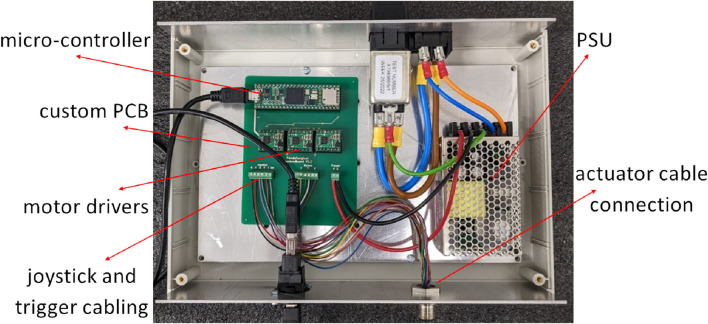
The control box housing all the electronics.

To keep the device lightweight, all electronics driving the motors and control interfaces, as well as the microcontroller implementing the control operation, are placed outside of the handheld controller. The motor drivers used for our novel prototype are the DRV8876 single brushed DC-motor driver carriers (Pololu, USA) with integrated current-threshold measuring. The motors were moving the robot-joints in velocity domain, until a current threshold was reached. The microcontroller interfacing with all motors and electronics was a Teensy 4.1 (PJRC, United states), with all these components resting in a custom-designed circuit board made with the V-One PCB printer (Voltera, CA). An opened control box, the cabling, as well as the electronics can be seen in Figure 8.

### 2.4 Experimental evaluation

#### 2.4.1 Workspace, structural integrity, and force delivery

During these experimental procedures, only the grasper and ring-curette end-effectors were investigated, since the ring-curette and dissector share the exact same joint design. The grasper end-effector, however, has a hollow middle channel for the DoF that actuates the grasper at the distal end.

To identify the joint-space of the robotic end-effector of the handheld instrument we used a protractor and moved the end-effectors to their joint limits, until the motor would stop outputting torque. We actuated the end-effectors on each axis individually, as well as simultaneously.

**FIGURE 9:**
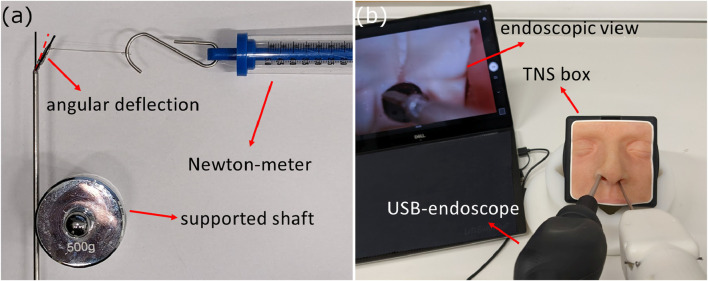
**(A)** Structural integrity test experimental setup, and **(B)** Phantom feasibility test experimental setup.

Then, to investigate the end-effectors’ structural integrity, the grasper and ring-curette were moved to a range of joint-spaces, namely, in [0,0], [±*joint limit*/2, 0], [0, ±*joint limit*/2], [±*joint limit*, 0], [0, ±*joint limit*], and [±*joint limit*, ±*joint limit*], and forces were applied with a Newton-meter and with the shaft supported at the tip of each end-effector in the *Y* and *Z* directions applying forces tangentially to the pitch and yaw robot joint axes respectively. The value at which angular deflection was noticed on the joint was recorded as the maximum force the end-effector can withstand at its tip when applied in that particular direction. This experimental setup can be seen in Figure 9A.

Finally, the two end-effectors were tested for their force-delivery capabilities with the use of the 6-axis F/T sensor Nano17 (ATI Industrial Automation Inc., CA). With their shaft supported, the end-effectors were actuated on each axis individually, pushing against the force sensor measuring the force at the point when the motor would stop outputting torque. This procedure was repeated 5 times, and the average measured force was kept as the maximum output force delivered in that direction. In the case of the grasper end-effector, its grasping force was also measured with the use of a force-sensitive thin film sensor and an Arduino Uno microcontroller (Arduino AG, IT).

#### 2.4.2 Phantom feasibility test

The first pre-clinical experiment aimed at validating the feasibility of our novel device. To do that, we simulated a tumor extraction procedure using the handheld robotic instrument inside the TNS box (UpSurgeon, IT), a phantom model for the transsphenoidal endonasal approach. This model was chosen because it has been validated as a potentially useful surgical skills training tool for its face, content, and construct validity Newall et al. (2022). The phantom feasibility test setup is shown in Figure 9B.

A silicon tumor was inserted in the pituitary fossa region, and in combination with a USB-endoscope, a non-clinician participant, familiar with the TNS box and the device, was tasked with extracting the tumor using the articulated grasper, the ring-curette, and finally the spatula dissector. The scope of the experiment was to preliminary investigate structural integrity when interacting with soft tissue.

#### 2.4.3 Cadaver pilot study

The scope of the second pre-clinical study was also exploratory, relying on qualitative surgeon feedback. During this study, the feasibility of the robotic device was investigated in terms of workspace exploration and tissue interaction. Additionally, its introduction into the surgical workflow was observed, alongside the device robustness and durability. Additionally, the standard equipment consisted of a 0^*o*^ neuroendoscope accompanied with its tele-pack stack (Karl Storz SE & Co. KG, DE), and an endoscopic pituitary instrument set (B.Braun, DE).

One expert neurosurgeon, one intermediate-level neurosurgical trainee, as well as four novices with no dedicated pituitary surgery experience, were recruited from a single neurosurgical unit. A pre-clinical study design was adopted to evaluate the robotic instrument alongside the use of standard endoscopic instruments on a single cadaver. The cadaver was placed supine. The head was positioned in the “conversational” position with the neck flexed and turned to the right, facing the surgeon. The head was draped and the nostrils were exposed.

The EETS including the durotomy was performed pre-task. The participants were instructed to navigate through the nasal passage in order to reach the sphenoid sinus with the use of standard endoscopic instruments and expose the pituitary gland. This was followed by the introduction of the robotic instrument, with the participants being instructed to enter and explore the pituitary fossa using the articulated robotic instrument and interact with soft tissue and bony structures.

Finally, after all 6 participants tried the robotic instrument, the expert and intermediate neurosurgeons tried the robotic endoscope end-effector, alongside the robotic instrument, both with and without the guidance of the standard rigid endoscope in order to visualize inaccessible areas.

To qualitatively evaluate the device performance, the surgeons filled-in a survey that included questions on structural integrity, precision, increased dexterity, force-delivery, easy of use and comfort.

## 3 Results

### 3.1 Workspace, structural integrity, and force delivery

**FIGURE 10:**
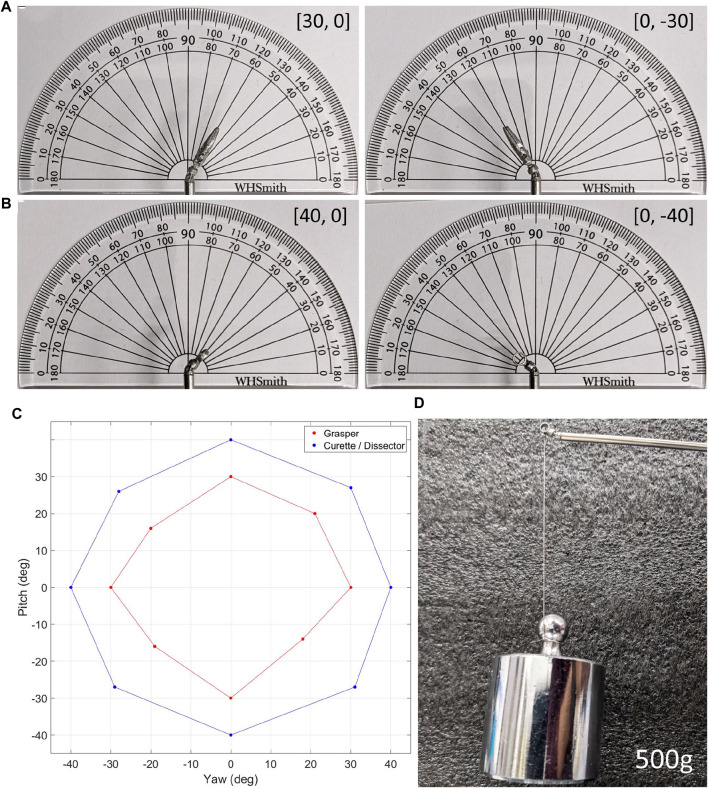
**(A)**. The grasper end-effector at two different joint-space limits, **(B)**. The ring-curette end-effector at two different joint-space limits, **(C)**. The overall joint-spaces of both end-effectors, and **(D)**. The ring-curette end-effector maintaining its pose holding a 500 g weight.

The joint limits of the grasper and ring-curette end-effectors when each DoF was actuated individually can be seen in Figures 10A,B respectively.

For the grasper end-effector, when each DoF was actuated individually with the other DoF staying locked at 0^*o*^, the joint limits were ±30^*o*^ for both DoF. In the case of the ring-curette, however, the same joint-limits were ±40^*o*^. In all end-effectors, when both DoF were actuated simultaneously, the joint limits were reduced. The overall joint-spaces of the end-effectors are evident in Figure 10C.

The maximum forces that the grasper and ring-curette end-effectors could withstand in a wide range of joint-spaces without noticeable angular deflection, maintaining, thus, their pose, are laid out in Table 1. The grasper end-effector could withstand a maximum force of 2*N* when both joints were actuated to their joint-limits and this force was applied on the *Z*-axis. The minimum force, 0.5*N* in the *Y*′ direction, was recorded at the end-effector’s neutral position. The maximum force withstood by the ring-curette was 5*N* in either *Z* − *Z*′ direction when the pitch joint was individually actuated and the minimum was 1*N* in the *Y* direction, both at the (−20, 0) joint-space, as well as at the (20, 0). The maximum force of 5*N* achieved during this experimental procedure is replicated in Figure 10D, where the ring-curette maintains a 500*g* weight.

**TABLE 1:** The measured forces applied at the tip of the end-effectors in the ZZ’ and YY’ directions while at discrete joint-spaces, at which angular deflection was noticeable. These forces represent the maximum forces each end-effector can withstand at its tip when applied in that particular direction.

Robotic grasper	(-30,0)	(-15,0)	(0,0)	(15,0)	(30,0)	(0,-30)	(0,-15)	(0,15)	(0,30)	(21,20)	(18,-14)	(-19,-16)	(-20,16)
Joint-space (yaw,pitch)													
***F***_***Z***_ ***(N)***	0.9	0.8	1	0.8	0.8	1.5	0.8	1	1.7	2	1.5	0.6	1.1
***F***_***Z′***_ ***(N)***	1.2	0.6	0.9	0.6	0.7	1.3	1	0.8	1.5	1.5	1.7	0.8	1.2
***F***_***Y***_ ***(N)***	1.3	0.5	0.7	0.7	1.5	1	0.6	0.8	1.1	1.6	1.4	1.1	0.9
***F***_***Y′***_ ***(N)***	1.2	0.7	0.5	0.7	1.2	0.8	0.6	0.6	1	1.4	1.2	1.2	1.4

Robotic curette	(-40,0)	(-20,0)	(0,0)	(20,0)	(40,0)	(0,-40)	(0,-20)	(0,20)	(0,40)	(31,27)	(31,-27)	(-29,-27)	(-28,26)
Joint-space (yaw,pitch)													
***F***_***Z***_ ***(N)***	2.5	2	1.6	1.4	2.5	3.5	1.3	3.5	5	1.8	2.5	1.5	1.8
***F***_***Z′***_ ***(N)***	2.3	1.5	1.6	1.2	3	3	1.6	1.8	5	2	2.2	2	2.3
***F***_***Y***_ ***(N)***	2	1	1.5	1	2	1.8	1.1	1.7	2.5	1.1	1.3	2	2
***F***_***Y′***_ ***(N)***	1.5	1.2	1.5	1.2	1.8	1.9	1.2	2	2	1.3	1.6	2.2	1.6

Finally, and regarding the force delivery capability of the end-effectors, the measured forces when the end-effectors were moved to the individual DoF limit joint-spaces are shown in Table 2. Both end-effectors showcased similar force-delivery capabilities in all different directions, with the grasper end-effector having the lower force thresholds between the two. The grasping-force recorded with the force-sensitive sensor for the grasper end-effector had a maximum value of 2*N*.

**TABLE 2:** The forces measured by the F/T sensor when the end-effectors were moved to discrete joint-spaces.

Robotic grasper	(-30,0)	(30,0)	(0,-30)	(0,30)
Joint-space (yaw,pitch)				
***F(N)***	0.78	0.66	0.86	0.79

Robotic curette				
Joint-space (yaw,pitch)	(-40,0)	(40,0)	(0,-40)	(0,40)
***F(N)***	1.34	1.27	1.24	1.29

The data presented in Table 1 showcase the structural integrity of the end-effector, namely, how much force could be applied on the end-effector, with the end-effector maintaining its pose. This test represents tasks such as tool insertion and removal, tissue retraction, and bony tissue collision, where the end-effector needs to withstand forces. The data presented in Table 2 showcase the force-delivery capability of the end-effector, namely, the amount of force the end-effector could apply. This test represents tasks such as tissue massaging and manipulation. While the two tables cannot be directly correlated, they together inform us of the robustness and strength of the miniature end-effector.

### 3.2 Phantom feasibility test

**FIGURE 11:**
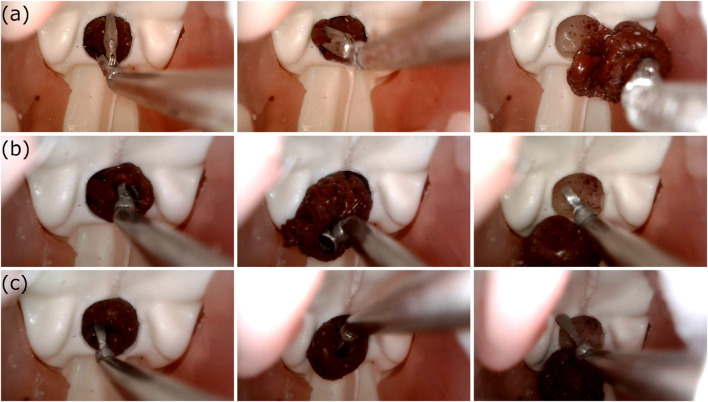
The view from the USB-endoscope during the phantom feasibility study where the silicone tumor at the pituitary gland region was removed with the use of **(A)**The robotic grasper, **(B)**The robotic ring-curette, and **(C)**The robotic dissector.

The participant managed to remove the tumor with all three end-effectors in 14*sec* with the grasper, 33*sec* with the ring-curette, and 26*sec* with the dissector. Endoscopic view frames extracted from the USB-endocope during the experiment are depicted in Figure 11.

### 3.3 Cadaver pilot study

**FIGURE 12:**
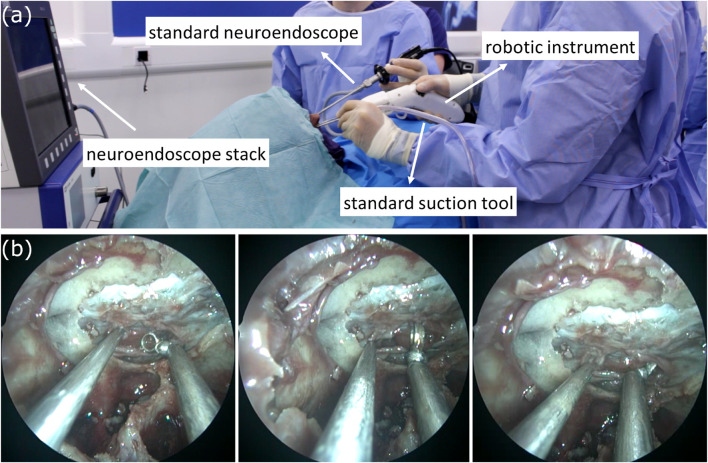
**(A)** The surgical setup with the introduction of the novel robotic instrument, and **(B)** (left to right) A standard suction tool, and the robotic curette in different poses interacting with soft tissue.

Figure 12A presents the robotic tool used in the cadaveric specimen, with Figure 12B showing the standard endoscope view of the novel robotic curette interacting with the sellar anatomy. To qualitatively evaluate the feasibility of the device, feedback regarding the dexterity, force delivery, structural integrity of the robotic device, and overall user experience was obtained through a post-task questionnaire.

The questions and surgeon replies are presented in Table 3. All participants reported the articulated robotic instrument provided greater dexterity than existing tools and maintained its structural integrity during the task. All six participants also felt the instrument applied sufficient force when pre-positioned at an angle in free space. However, the inability of sufficient forces to be applied during the movement of the instrument and the lack of precise movements were noted by all participants. In terms of user experience, all six participants reported the instrument was intuitive and comfortable to use, with the noise and wires not having an impact on workflow.

**TABLE 3:** The post-cadaver study questionnaire, and the surgeon replies.

Question	Yes	No
Does the robot maintain its pose when pressing against soft and boney tissue?	6	0
Does the robot allow for precise movements?	0	6
Does the robot allow for increased dexterity?	6	0
Does the robot apply sufficient forces when angled?	6	0
Does the robot apply sufficient forces while in motion?	0	6
Is the robot easy to use?	6	0
Is the robot comfortable to use?	6	0

**FIGURE 13:**
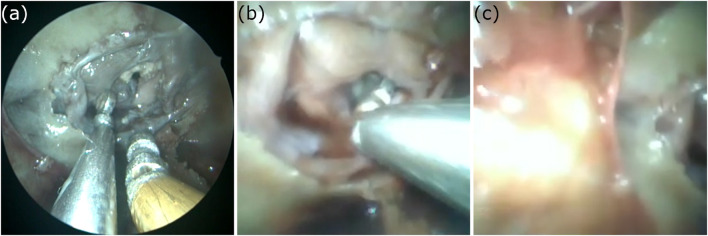
**(A)** Concurrent usage of the robotic ring-curette and the robotic endoscope, while in visual guidance from the standard neuroendoscope, **(B)** View of the sellar anatomy using the articulated endoscope, in its initial pose, and **(C)** View of the sellar anatomy using the articulated endoscope, having it actuated on the yaw axis.

Finally, regarding the concurrent usage of an articulated instrument and articulated endoscope, the takeaway was that while promising, this work was preliminary. The camera quality was not sufficient, the depth perception of the articulated visual elements is challenging and, the best usage of such a device would most likely be alongside a standard endoscope that would ensure the articulated endoscope elements don’t collide with soft tissue. The concurrent usage of the two robotic tools is shown in Figure 13A, with Figures 13B,C depicting two views from the articulated endoscope.

## 4 Discussion

In this work, we present a novel handheld robotic system for endoscopic neurosurgery, with an ergonomically designed handheld controller, and a series of 3 *mm* tendon-driven spherical joint robotic end-effectors.

The spherical-joint design and routing-mechanism of this system allowed for a significant workspace increase compared to rigid instrumentation with a ± 30° joint-limit for the articulated grasper, and ± 40° for the ring-curetted and dissector. This difference in joint-space is a result of the wider middle segment of the grasper, still within the 3 *mm* diameter, that allows a path for the grasper DoF tendons. These limits are smaller than other similar-sized end-effectors, such as the ones presented in Kwon et al. (2018) and Arata et al. (2019). However, a number of advantages this design offers, namely, the miniature size of the 3 *mm* robots, the concentration of both DoF on the same point, and the handheld nature of the device, suggest significantly increased dexterity and ability to navigate within the confined spaces of the EEEA.

In Bekeny et al. (2013), it is recorded that average forces during soft tissue excision of pituitary lesions are in the 0.1*N*–0.5*N* range in the *X*, *Y*, and *Z* directions. Maximal forces tended to occur in the *Z* direction, especially with bony collisions and peak at 2.12*N*. Both end-effectors could comfortably withstand the maximum force of 0.5*N* associated with the soft-tissue excision phase of the operation, as showcased during the structural-integrity tests, with the ring-curette having the better capability to withstand even the maximum force associated with bony collisions. The grasper would not be able to withstand 2*N* of force in most poses and needs further development. The structural-integrity discrepancy between the two end-effectors, as well as the clear limitation of the grasper in that front, we believe is a result of the tendon pre-tensioning value of the two end-effectors. It is possible that using the same pre-tensioning methodology and value for both designs was not a sufficient approach, and in future work, we will investigate the most appropriate pre-tensioning values for the grasper end-effector.

During this engineering test-set, our end-effectors showcased the least capability in terms of force-delivery. While there are anticipated limitations due to the miniature size of the robot-joint, there is a number of actions we intend to take to increase force-delivery capability. The mechanical backlash and friction in the end-effector housing will be reduced by replacing current materials with better surface-finish materials, and the motors and their gearboxes will be replaced with higher continuous torque motor assemblies.

A limitation of this current system development is the absence of kinematic and dynamic modelling, with this analysis being out of scope for this initial study. In this paper, we set out to implement a fully-functional robotic prototype that would be controlled via open-loop control, to answer the fundamental questions of dexterity, robustness and clinical feasibility. In future work, we intend to simulate the kinematics and dynamics of the miniature end-effectors to improve accuracy and robustness.

The pre-clinical evaluation re-iterated these findings. The phantom feasibility test suggested increased dexterity and adequate robustness for tumor extraction, with the limitation that the silicone tumor was conveniently located in the skull-base cavity, and thus, workspace exploration could not be adequately investigated. For future validation, we plan on customizing the phantom physiology to allow for an operative workspace increase, more similar to the expanded endoscopic endonasal approach.

Finally, during the cadaver pilot study, the received surgeon feedback allowed us to investigate the current development stage of the robotic system. The favorable results on workspace exploration, dexterity, structural integrity and ease-of-use, suggest that future development should focus on the study limitations, namely, the imprecise control, and the force-delivery capabilities. The current open-loop control methodology, will be replaced with a more sophisticated position-domain closed-loop control in future work that will map the robot joints movements to the control interfaces.

## 5 Conclusion

In this work, we present a novel handheld robotic system for endoscopic neurosurgery, using the EEEA as an exemplar. The system is comprised of a series of 3 *mm* detachable articulated end-effectors, coupled with an ergonomically-designed handheld controller. To further showcase the modularity of this joint design, the end-effector was altered to accommodate an articulated endoscope. The system was put through a series of engineering tests to understand its workspace capabilities and robustness. Finally, its clinical feasibility was evaluated during two pre-clinical studies, a phantom-feasibility test, and a cadaver pilot study. Results from the experimental and pre-clinical validation cycles suggested dexterity and workspace increase with reliable soft-tissue interaction, with more development required to improve the force-delivery capability of the system, as well as its control precision. Future work will aim to further improve the device with a clear goal for *in vivo* clinical validation and surgical adoption.

## Data Availability

The original contributions presented in the study are included in the article/Supplementary Material, further inquiries can be directed to the corresponding author.
